# Heat stress affects dairy cow performance via oxidative stress, hypothalamic–pituitary–adrenal axis, gut microbiota, and multi-dimensional mitigation

**DOI:** 10.3389/fvets.2025.1686241

**Published:** 2025-10-17

**Authors:** Qiang Zhang, Lulu Yang, Yunhan Li, Pengbo Gu, Riguleng Si, Lin Zhu, Wenguang Zhang

**Affiliations:** College of Animal Science, Inner Mongolia Agricultural University, Hohhot, China

**Keywords:** heat stress, milk production efficiency, nutritional regulation, genetic breeding, dairy cows

## Abstract

Against the backdrop of global warming, heat stress has become one of the greatest challenges facing the dairy industry. This review systematically summarizes the multi-dimensional impacts of heat stress on dairy cows and corresponding mitigation strategies under global warming. It covers the evaluation indicators (temperature-humidity index, physiological and behavioral indicators) and classification of heat stress. It analyzes the negative effects on dairy cows’ reproductive performance (e.g., oocyte and sperm damage, hormonal disorders, impaired fetal development) and production performance (e.g., reduced milk yield, deteriorated milk composition). These effects are mediated by physiological mechanisms such as oxidative stress imbalance, hypothalamic–pituitary–adrenal axis (HPA axis) activation, cellular structural damage, altered gene expression, and disrupted host-gut microbiota interactions. Additionally, the review integrates interdisciplinary mitigation strategies including environmental optimization, nutritional regulation, genetic breeding, and intelligent monitoring. It provides theoretical and practical references for constructing a sustainable heat stress prevention and control system.

## 1 Introduction

As a crucial component of global agriculture, livestock farming is facing severe challenges posed by climate change (1). In recent years, with the gradual rise of global temperatures, heat stress has become one of the main factors affecting the production level and welfare of dairy cows (2). Heat stress impairs cow health, redirects energy toward heat dissipation, thereby causing a sharp decline in milk yield and economic losses. Annual economic losses due to heat stress amount to billions of US dollars (3, 4).

As homeothermic animals, dairy cows thrive within a temperature range of 10–20 °C (5). When the temperature-humidity index (THI) exceeds a specific critical value, the impacts of heat stress begin to manifest (4). Due to long-term artificial selection, modern high-yielding Holstein cows have an increased metabolic rate, with milk yield rising by 300% (relative increase) compared to that of dairy cows 1950, significantly improving economic efficiency. However, this has also lowered their heat tolerance threshold (6). The contradiction between “high productivity and heat sensitivity” makes heat stress a key factor restricting the sustainable development of the dairy industry (7). Moreover, significant differences in heat tolerance exist among different dairy cow breeds (8). Compared with Holstein cows, Jersey cows possess thinner hair coats (0.3–0.4 mm vs. 0.5–0.6 mm in Holsteins) and 15% higher skin capillary density (relative increase) (8). This enables more efficient surface heat dissipation and stronger heat tolerance. Local breeds such as Luxi Yellow Cattle and Mongolian Cattle have adapted to temperate to subtropical climates over the long term (9). They exhibit a higher heat tolerance threshold and smaller fluctuations in physiological indicators under heat stress (9). Their dry matter intake (DMI) reduction rate of only 5–8% compared to 10–15% in Holsteins (6).

The dairy industry is widely distributed geographically, and differences in environmental conditions across climate zones and feeding models significantly impact dairy cows’ heat stress responses (10). Globally, dairy cows are mainly reared in three major climate zones (11). Tropical regions (e.g., Kenya): The average daily temperature often exceeds 22 °C, and THI remains above 69 throughout the year, resulting in persistent heat stress (12). Subtropical regions (e.g., the Yangtze River Basin in China, northern Italy): THI fluctuates between 65 and 85 in summer, with obvious seasonal heat stress (June–September). High humidity (RH > 70%) accompanies this, exacerbating difficulties in surface heat dissipation (1). Temperate regions (e.g., Inner Mongolia in China, northern United States): Short-term heat stress (THI 72–78) only occurs in summer (July–August) (13). Insulation measures are required in winter to avoid alternating impacts of cold stress and heat stress on dairy cow physiology (9).

Meanwhile, large-scale intensive farms mostly adopt closed barns. Although equipped with cooling equipment such as ventilation and sprinkler systems (resulting in strong heat stress regulation capabilities), the barn density (10–15 cows per 100 m^2^) may exacerbate group heat accumulation (14). In contrast, free-range/semi-free-range farms (e.g., grasslands in northern China) rely on natural shading such as trees and sheds. Their cooling equipment coverage rate is less than 30%, and dairy cows are exposed to the natural environment for longer periods. This increases the vulnerability of their heat stress risk to weather fluctuations (15). Additionally, different feed supply models (e.g., total mixed ration (TMR) in intensive farming, grazing + supplementary feeding in free-range systems) can alter the heat stress tolerance of dairy cows. This is achieved by influencing their energy metabolism status—for instance, high-energy diets may increase metabolic heat production and enhance heat stress sensitivity (16).

**Figure 1:**
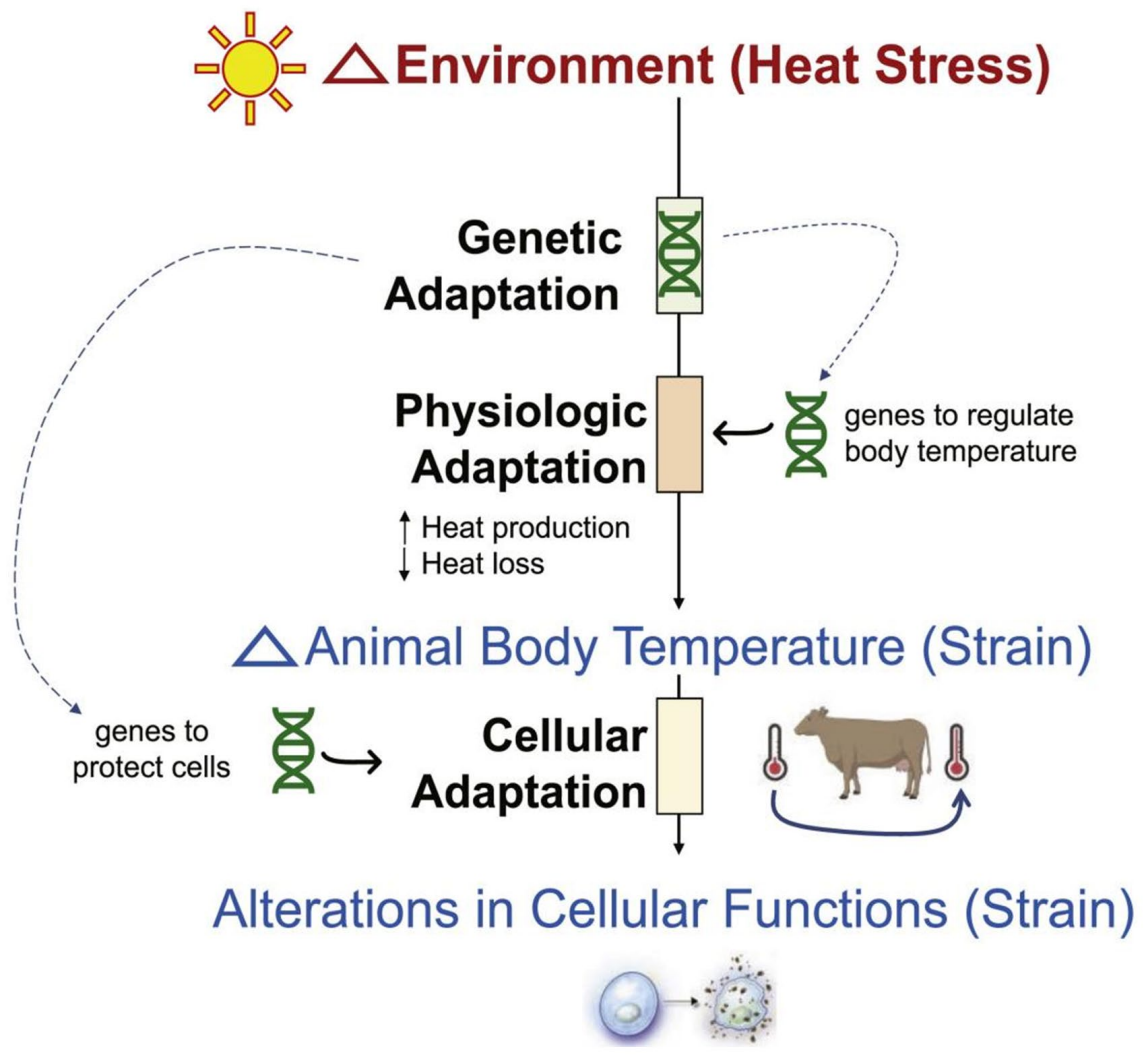
Impact of heat stress on dairy cows. In a heat stress environment, animals respond to changes in body temperature through genetic adaptation, physiological adaptation (regulating heat production and heat loss), and cellular adaptation, which in turn affect changes in cellular functions. Among them, genetic adaptation involves genes that regulate body temperature and genes that protect cells.

The impact pathway of heat stress on dairy cows can be summarized as follows: environmental triggers (e.g., elevated THI) induce physiological changes. These changes then cause molecular and cellular damage, ultimately leading to reduced reproductive performance and milk yield (17). The specific mechanism is shown in Figure 1 (18).

Heat stress is a systemic physiological disorder caused when ambient temperature exceeds an animal’s maximum heat dissipation capacity, resulting in impaired thermoregulation (13). There are three main evaluation indicators:

①Temperature-Humidity Index (THI), calculated as THI = 0.81 × T + (0.99 × T − 14.3) × (RH/100) + 46.3, where T is the dry-bulb temperature (°C) and RH is relative humidity (%) (19).
②Physiological indicators: When respiratory rate exceeds 60 breaths/min, salivation increases twofold to enhance evaporation of moisture from the body surface (20).
③Behavioral indicators: Reduced lying time (<12 h/d), decreased feeding frequency, and open-mouth breathing in over 30% of the herd (21).

**Figure 2:**
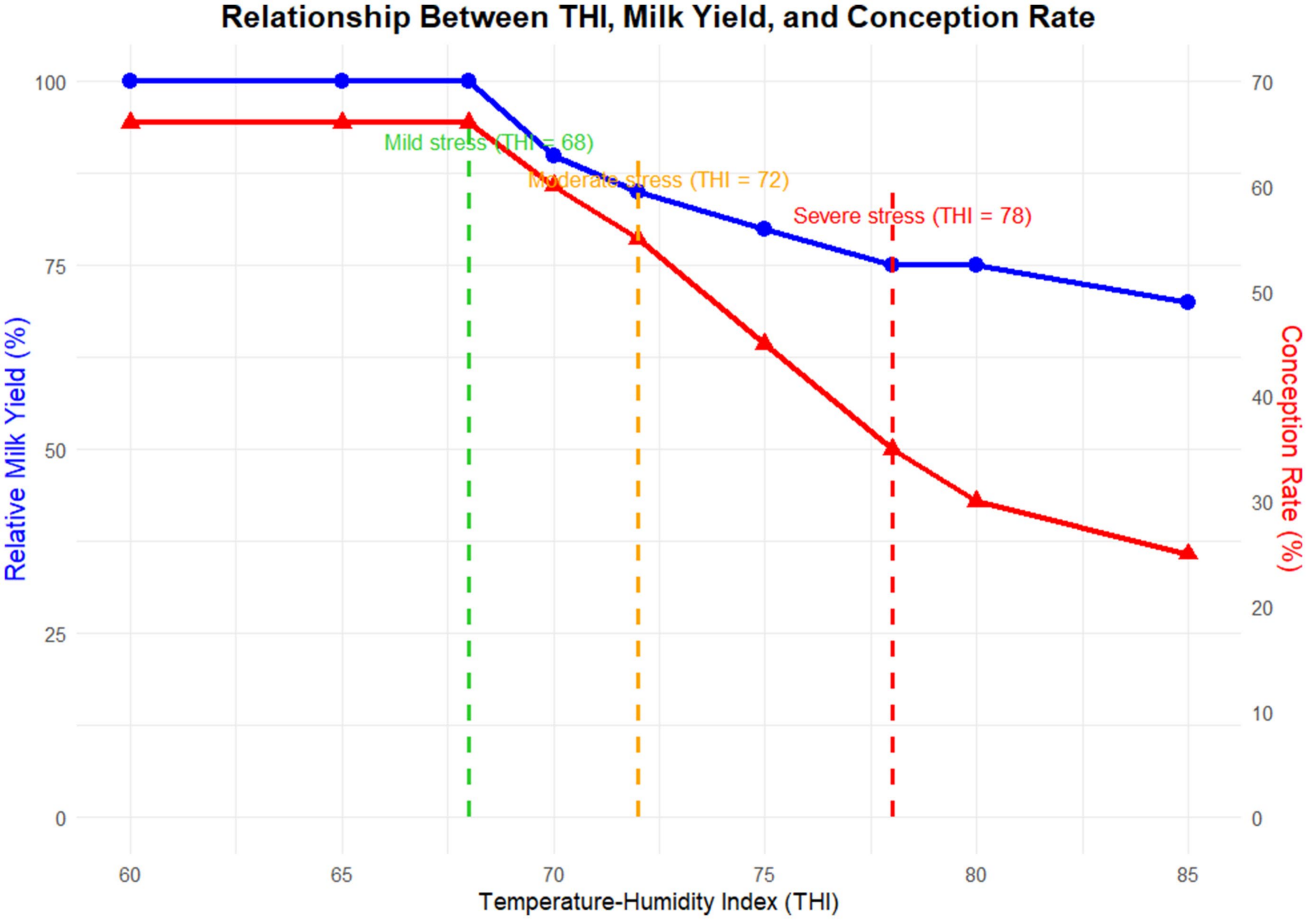
Line graph showing the association between increased THI and decreased milk yield and conception rate. Relationship between Temperature—Humidity Index and cows’ relative milk yield (blue line) and conception rate (red line): As the Temperature—Humidity Index rises (entering mild, moderate, and severe stress), both relative milk yield and conception rate decrease continuously.

THI heat stress grading threshold criteria: When THI < 68, cows are in a state of no obvious stress, within the comfortable temperature range of 10–20 °C. Their thermoregulatory mechanisms are stable and indicators such as respiratory rate and rectal temperature remain normal (22). When 68 ≤ THI < 72, cows experience mild stress, showing initial stress signs (slightly reduced feed intake, potentially decreased lying time and increased standing activity). Milk components (milk protein, milk fat) show no significant changes, but energy metabolism begins to shift toward heat dissipation (23). When 72 ≤ THI < 78, cows suffer moderate stress. Respiratory rate increases significantly (>60 breaths/min), salivation reaches 1.5–2 times the normal level, rectal temperature rising above 39.5 °C. Daily milk yield decreases (1.3–6.7 kg/d), and total summer milk yield 15–25% lower than in winter (relative decrease) (see section 4.1) (24). When THI ≥ 78, cows undergo severe stress. Rectal temperature exceeds 40 °C, and respiratory rate may exceed 80 breaths/min. This leads to respiratory alkalosis (blood pH > 7.5) and significantly deteriorated milk components (e.g., reduced milk protein rate) (see section 4.2) (25). When THI > 80, cows face extreme stress, with physiological and reproductive crises. Permanent damage to milk production capacity may occur, and prenatal heat stress affecting offspring through epigenetic mechanisms (e.g., poor mammary development in adult calves with 20% fewer alveoli) (relative decrease) (26, 27). These classifications provide a precise basis for heat stress management—for example, initiating active cooling measures when THI > 72 and strengthening reproductive intervention and nutritional regulation when THI > 78 (25). The relationship between THI and milk yield/conception rate is shown in Figure 2. Although the multi-faceted impacts of heat stress on cows’ physiological mechanisms, production performance, and reproductive capacity have been confirmed, further research is needed on its long-term consequences and effective management technologies (28).

## 2 Analysis of physiological mechanisms of heat stress

**Figure 3:**
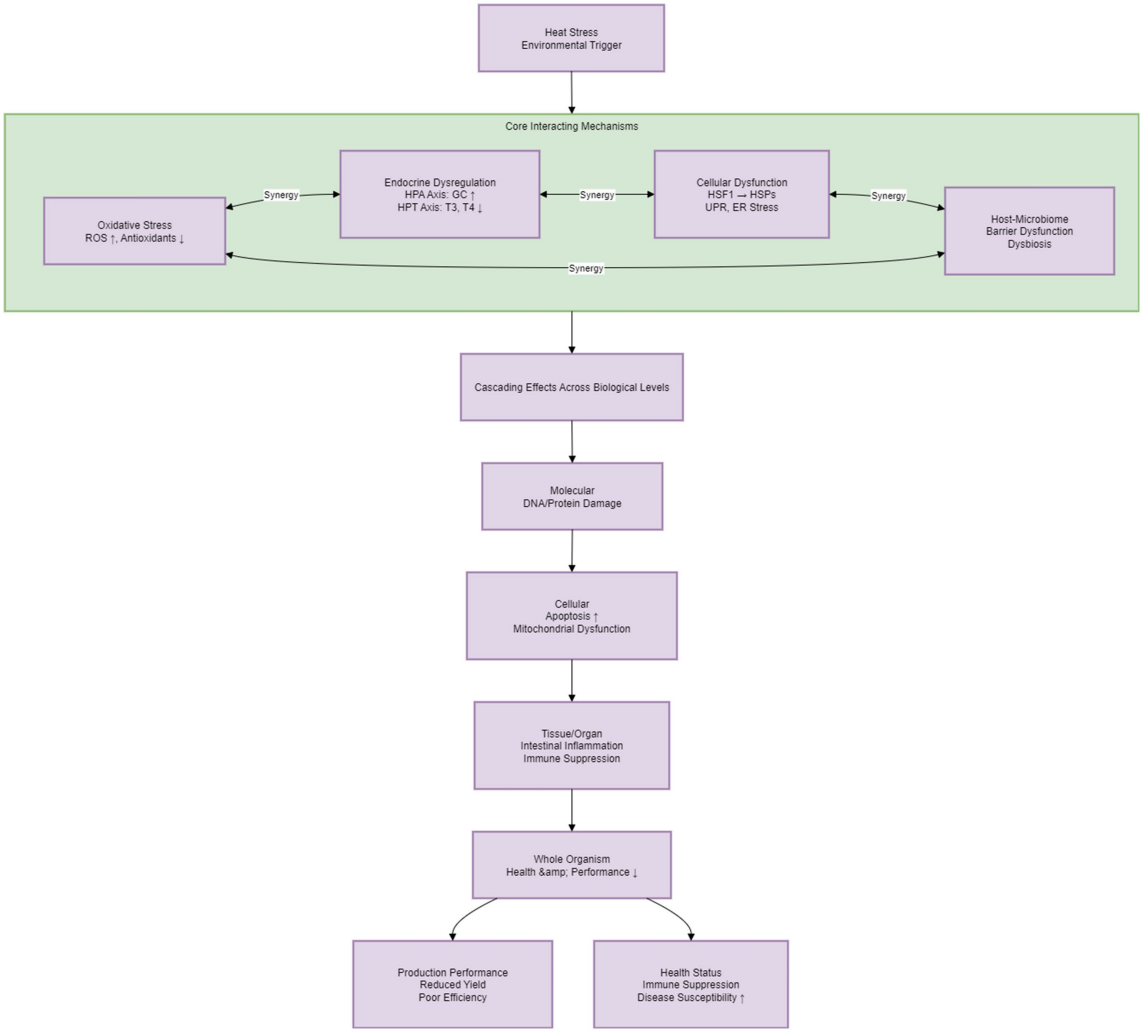
Physiological impacts of heat stress on dairy cows. Heat stress triggers core dysfunctions (oxidative stress, endocrine/cellular/gut-microbiome disorders) that interact. Damage cascades from molecular (DNA/protein harm) → cellular (apoptosis, mitochondrial issues) → tissue/organ (inflammation, immune suppression). Finally, whole-organism health, reproduction, and production decline (lower yield, weaker immunity, more disease).

The impact pathway of heat stress on dairy cows starts with environmental triggers (e.g., elevated THI) (6). These triggers induce physiological changes, which then cause molecular and cellular damage, ultimately leading to reduced reproductive performance and milk yield (18). This process is characterized by the core feature of “systemic disorder-targeted damage”—it specifically affects health and production performance from the molecular to the organismal level (6). This occurs through the synergistic effects of oxidative stress, endocrine regulation, cellular function damage, and host–microbe interaction (Figure 3) (29).

### 2.1 Imbalance between oxidative stress and antioxidant system

Heat stress first disrupts the oxidative balance of dairy cows and activates the hypothalamic–pituitary–adrenal (HPA) axis, with both factors synergistically exacerbating physiological damage (17).

On one hand, heat stress causes abnormal function of the mitochondrial electron transport chain, increasing the production of reactive oxygen species (ROS) such as superoxide anions and hydrogen peroxide by 2–3 times (30). This accumulation exceeds the scavenging capacity of antioxidant enzymes, including superoxide dismutase (SOD) and catalase (CAT) (31). This imbalance triggers a chain reaction: lipid peroxidation increases malondialdehyde (MDA) levels; protein glycosylation damages whey protein; DNA strand breaks raise 8-hydroxy-2′-deoxyguanosine (8-OHdG) levels by 50% (relative increase) (6). Additionally, ROS activate the JNK/p38 mitogen-activated protein kinase pathway, increasing the apoptosis rate of mammary epithelial cells from 5 to 20% (absolute proportion) (32). Although the body can enhance protein repair capacity by upregulating heat shock proteins (e.g., HSP70 expression increases fivefold within 2 h of heat stress) and activate the Nrf2 signaling pathway to promote antioxidant enzyme gene transcription and initiate compensatory mechanisms, heat stress lasting more than 24 h gradually disables these mechanisms (6). On the other hand, excessive ROS can cross the blood–brain barrier of dairy cows, activate the TRPV1 channel in the paraventricular nucleus of the hypothalamus, stimulate the secretion of corticotropin-releasing hormone (CRH), and trigger the HPA axis cascade reaction (17). The secretion of adrenocorticotropic hormone (ACTH) from the anterior pituitary increases to 2–3 times the normal level (33). Cortisol synthesized by the adrenal cortex rises significantly: after 72 h of continuous heat stress, plasma cortisol concentration reaches 1.8 times the baseline and fails to restore its circadian rhythm at night, disrupting the negative feedback balance of the HPA axis (34, 35). High cortisol levels also inhibit the immune function of dairy cows (36): the proliferative capacity of T lymphocytes decreases by 40–50% (relative decrease), and the titer of B cell-specific antibodies decreases by more than 30% (relative decrease) (37, 38). Meanwhile, the incidence of mastitis increases by 2–3 times (39). Histone deacetylation inhibits the synthesis of pro-inflammatory factors such as IL-6 and TNF-*α*, and when combined with endotoxemia caused by intestinal barrier damage, it leads to “chronic low-grade inflammation”—a state that further exacerbates the decline in milk yield and reproductive dysfunction (39, 40).

### 2.2 Cellular and gene expression changes: targeted effects of structural damage and molecular regulation

Based on the physiological disorders induced by oxidative stress and HPA axis activation, heat stress further targets and impairs the core physiological functions of dairy cows (e.g., lactation, reproduction) from the cellular structural to the gene expression level (30). Such damage significantly intensifies as stress severity increases when THI ≥ 72 (41, 42).

At the cellular level, mitochondrial damage is the most prominent: decreased mitochondrial membrane fluidity and increased permeability impair the electron transport chain, reducing ATP production (directly associated with insufficient energy for lactation) (43). Simultaneously, the expression of the mitochondrial fission-related gene Drp1 is upregulated, while the expression of the fusion-related gene Mfn2 is downregulated, leading to mitochondrial fragmentation (31). Excessive ROS produced by abnormal mitochondrial function further damages mitochondrial DNA, ultimately forming a vicious cycle of “structural damage → reduced function → ROS accumulation → further damage” (30).

In addition, under heat stress, the endoplasmic reticulum (ER) of dairy cows accumulates large amounts of unfolded proteins, triggering the unfolded protein response (UPR)—a self-protective mechanism that alleviates ER stress by inhibiting protein synthesis and enhancing ER-associated degradation (44). If heat stress persists beyond the ER’s self-repair capacity, the PERK/eIF2α/CHOP pathway is further activated, ultimately inducing cell apoptosis (45).

**Figure 4:**
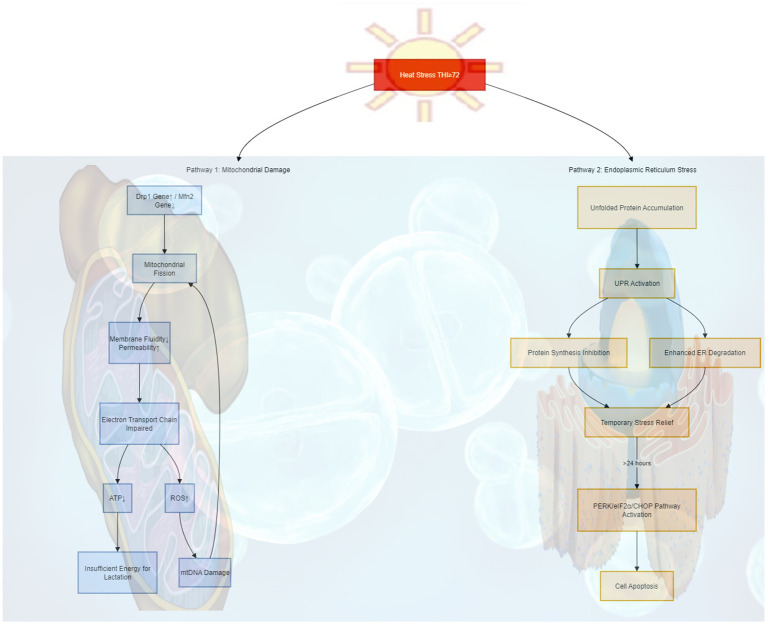
Damage of heat stress to cells. Heat stress damages cells mainly through two pathways: one is causing abnormal mitochondrial fission and impaired function, leading to insufficient lactation energy; the other is causing the accumulation of unfolded proteins in the endoplasmic reticulum, which, after a short-term relief, eventually activates pathways to induce cell apoptosis.

At the gene expression level, heat stress regulates gene expression patterns through multiple pathways (46). Upon activation, heat shock transcription factors (HSFs) bind to the promoter regions of heat shock genes, promoting the transcription and expression of heat shock proteins (e.g., HSP70, HSP90) and related genes to maintain cellular protein homeostasis (47). Additionally, heat stress alters gene expression profiles through various epigenetic mechanisms, including DNA methylation (e.g., increased methylation levels of the IGF1 gene promoter in mammary tissue), histone modification (e.g., increased H3K27ac levels, a modification marker associated with the activation of gene promoter regions), and non-coding RNA regulation (48). For the specific action pathways of the aforementioned mitochondrial damage and endoplasmic reticulum (ER) stress, please refer to Figure 4.

The role of microRNAs (miRNAs) in dairy cow heat stress has been initially clarified (49). For example, miR-25 expression increases in the mammary tissue of heat-stressed dairy cows, exacerbating mammary inflammation by targeting the JNK gene, while miR-181d affects the immune stress response by regulating IL1β expression (46). Beyond miRNAs, the potential regulatory roles of long non-coding RNAs (lncRNAs) and circular RNAs (circRNAs) have gradually attracted attention (50). In studies related to cattle, lncRNAs can regulate the transcription efficiency of heat shock genes (e.g., HSP70) by binding to their promoter regions, thereby enhancing cellular heat tolerance (51). CircRNAs can act as “miRNA sponges” to sequester stress-related miRNAs (e.g., sequestering miR-181d to relieve its inhibition of IL1β and maintain immune balance) (49). These findings suggest that non-coding RNAs may play similar regulatory roles in dairy cow heat stress, although research specific to dairy cows remains limited (52). Under heat stress, various key biomarkers in dairy cows undergo specific changes. These changes not only reflect heat stress’s impacts on cellular functions, immune status, and production- and reproduction-related physiological processes but also provide a molecular basis for assessing heat stress severity and investigating its mechanisms (46). The specific changes and functional effects are shown in Table 1.

**Table 1:** Changes and functional effects of key biomarkers in dairy cows under heat stress.

Biomarker type	Specific biomarker	Change trend (relative value)	Functional effect	References
Heat shock proteins	Heat shock protein 70 (HSP70)	Increased 5-fold within 2 h of heat stress	Repairs denatured proteins, maintains the survival of mammary epithelial cells, and enhances the heat tolerance of these cells	(6)
Inflammatory factor genes	Interleukin-6 (IL-6)	Decreased by 40% under the action of cortisol	Inhibits inflammatory response, aggravates immunosuppression; reduces the activation of anti-infection defense lines	(39)
	Tumor necrosis factor-*α* (TNF-α)	Decreased by 35% under the action of cortisol	Inhibits inflammatory response, impairs innate immune clearance ability	
	Interleukin-1β (IL-1*β*)	Decreased by 28% under the action of cortisol; decreased under the regulation of miR-181d	Affects immune stress response; maintains immune balance	(34)
miRNAs	miR-25	Increased in mammary tissue	Targets the JNK gene, aggravates mammary inflammation; reduces milk quality	(46)
	miR-181d	Increased under stress conditions	Inhibits the expression of IL-1β, affects immune stress response	
Mitochondria-related genes	Drp1 (fission gene)	Increased under heat stress	Promotes mitochondrial fragmentation, reduces ATP production efficiency	(30)
	Mfn2 (fusion gene)	Decreased under heat stress	Inhibits mitochondrial fusion, aggravates the loss of mitochondrial function	
Growth and reproduction-related genes	Insulin-like growth factor 1 (IGF1)	In mammary tissue (increased promoter methylation) → decreased expression	Fetal stage: Affects mammary gland development (20% reduction in alveolar number); adult stage: Reduces milk yield by 8–12%	(60)
	Follicle-stimulating hormone receptor (FSHR)	Decreased by 30–40% during the follicular phase	Inhibits the proliferation of granulosa cells, hinders follicle development	(25)
Lactation-related genes	β-lactoglobulin	Decreased by 30–40% in mammary tissue	Reduces milk protein synthesis, lowers milk quality	(46)
	α-Casein	Decreased by 30–40% in mammary tissue	Reduces milk protein synthesis, lowers milk quality	
Oxidative damage markers	Malondialdehyde (MDA)	Increased under heat stress	Reflects the degree of lipid peroxidation, indicates the level of oxidative damage	(6)
	8-Hydroxy-2′-deoxyguanosine (8-OHdG)	Increased by 50% under heat stress	Reflects the degree of DNA oxidative damage, indicates cellular molecular damage	

### 2.3 Intestinal and nutritional metabolism regulation: host-microbiome interaction mechanisms

In addition to oxidative stress and cellular function damage, heat stress indirectly affects the nutritional metabolism and immune function of dairy cows by disrupting host-gut microbiota interactions (53). This mechanism, together with the aforementioned HPA axis activation and oxidative imbalance, exerts a synergistic effect, collectively exacerbating the decline in milk yield and reproductive disorders.

As key participants in host metabolism and immune regulation, gut microbiota undergo changes in their community structure and function, which form a dynamic regulatory network with host gene expression through signaling pathways, collectively affecting the adaptability of dairy cows to heat stress (17). Integrating host transcriptome and gut microbiome data can clarify the multi-dimensional mechanisms by which heat stress affects dairy cow production performance. This reveals the core role of host-microbiome interactions in heat stress regulation (54).

Studies have shown that dominant genera in the gut microbiome (e.g., Clostridium, 57 N15, Treponema) occupy hub positions in the interaction network (55). Further mechanistic analysis indicates that the microbiota may affect the host through two pathways: On one hand, they may upregulate the expression of metabolic regulatory genes (e.g., SLC22A1) to maintain energy balance; on the other hand, they may inhibit the expression of stress response genes (e.g., ENSBTAG00000024272), reducing heat adaptation capacity (53). This finding provides new evidence for expanding the HPA axis theory in dairy cows (56).

This interaction directly reduces rumen fermentation efficiency, decreasing dry matter intake (DMI) by 10–15% (relative decrease) (57). Simultaneously, the production of microbial metabolites (e.g., short-chain fatty acids) decreases, further exacerbating negative energy balance—directly associated with reduced milk yield and disrupted reproductive hormones (57). Additionally, the combination of Clostridium abundance and the expression of cytokine pathway genes (IL-6, TNF-*α*) can serve as a heat stress biomarker, potentially reducing heat stress-related milk yield decline by 15–20% (relative decrease) (53). At the application level, the multi-component biomarker system proposed in the study (e.g., combining Clostridium abundance with cytokine pathway gene expression) offers new possibilities for the precise management of livestock (55).

### 2.4 Summary of the association between physiological mechanisms and dairy cow production/reproductive performance

Against the backdrop of global warming, the negative impacts of heat stress on dairy cow production performance and reproductive performance have become a key issue restricting the sustainable development of the dairy industry (25). Studies show that heat stress does not act on dairy cows through a single pathway, but exerts comprehensive effects via the “cascade effect” of multiple physiological mechanisms (6). The core pathways of action and specific mechanisms are as follows.

From the perspective of specific mechanisms of action, the three major physiological mechanisms affect dairy cow performance at different levels: First, at the level of oxidative and endocrine responses, excessive activation of the hypothalamic–pituitary–adrenal (HPA) axis leads to elevated cortisol levels (58). This state of high cortisol not only inhibits the proliferation of mammary epithelial cells and reduces the expression of genes related to milk protein synthesis, directly causing a decrease in milk yield, but also disrupts mitochondrial function in oocytes and reduces sperm motility, thereby impairing gamete quality (59). Second, at the level of cellular and gene expression changes, apoptosis of mammary epithelial cells and reduced mitochondrial ATP production caused by heat stress directly result in lactation dysfunction; meanwhile, abnormal methylation of the insulin-like growth factor 1 (IGF1) gene hinders fetal mammary gland development (with a 20% relative decrease in alveolar number), leading to an 8–12% relative decrease in milk yield of offspring when they reach adulthood (see section 3.5) (60). Third, at the level of disrupted intestinal and nutritional metabolism regulation, heat stress exacerbates the decline in rumen function, and the reduction in dry matter intake (DMI) decreases the energy supply required for lactation, further deteriorating milk yield and quality (61).

In summary, these three physiological mechanisms do not act independently but exhibit a synergistic amplification effect. They seriously threaten the economic benefits of the dairy industry by reducing milk yield, decreasing conception rates, and posing long-term risks such as impaired production potential of offspring (62). At the same time, these clear pathways of action also provide clear core targets for the subsequent development of targeted heat stress mitigation strategies (e.g., regulating HPA axis activity, improving intestinal metabolism, and intervening in gene methylation) (6).

## 3 Impacts of heat stress on dairy cow reproductive performance

Heat stress exerts systemic impacts on dairy cows through physiological mechanisms such as inducing oxidative stress imbalance, overactivation of the hypothalamic–pituitary–adrenal (HPA) axis, cellular structural damage, abnormal gene expression, and disrupted host-gut microbiota interactions (53).

**Figure 5:**
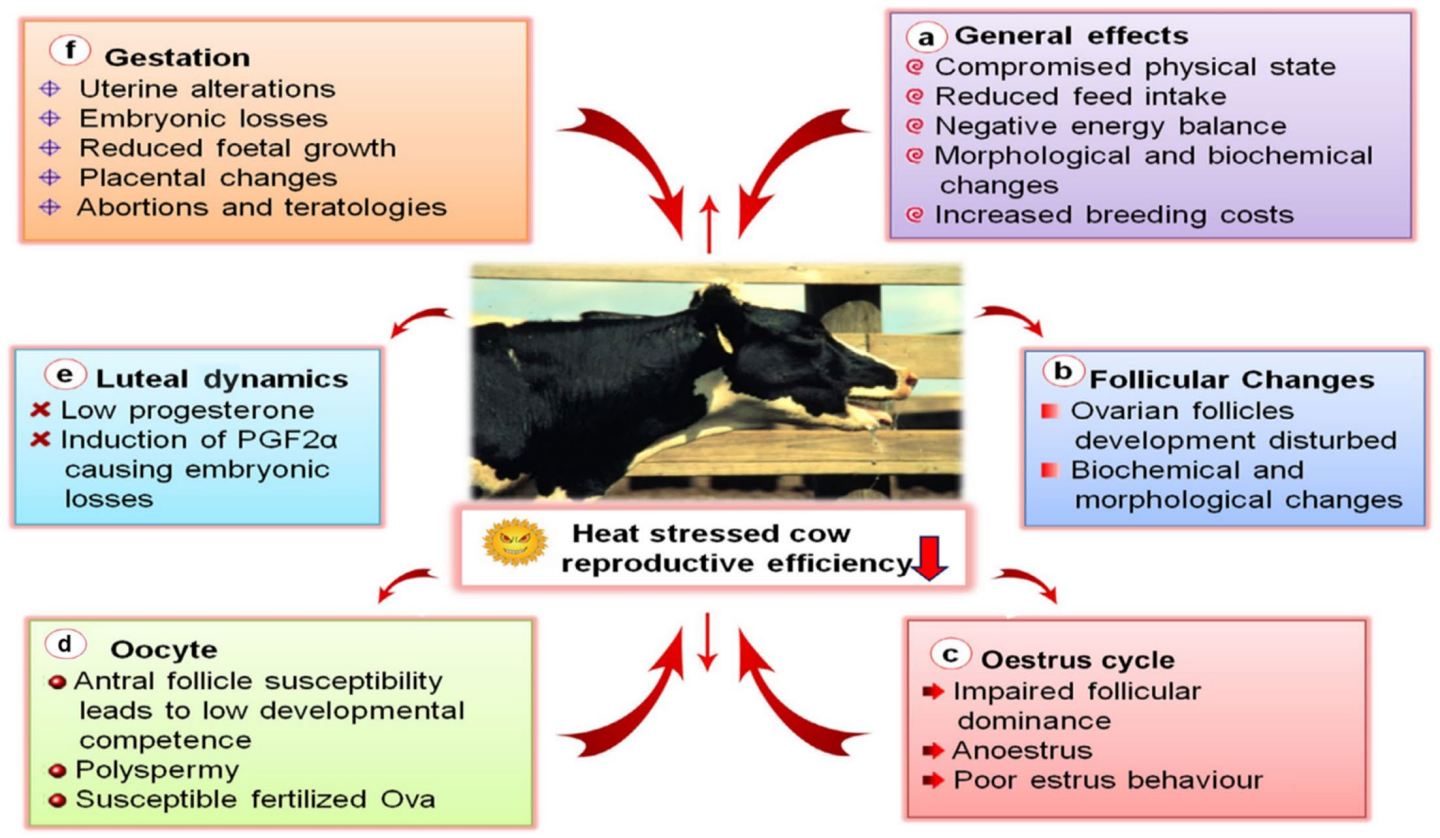
Impacts of heat stress on dairy cow reproduction, presented in box form (a–f). Multifaceted effects of heat stress on cows’ reproductive efficiency, including general impacts like impaired physical condition and reduced feed intake, as well as follicular changes, abnormal estrous cycles, altered luteal dynamics, and pregnancy—related damages (such as embryo loss, abortion, etc.).

Direct invasion of the reproductive system: From oocyte development, corpus luteum function maintenance, estrous cycle regulation to pregnancy process, it causes gamete damage (reduced oocyte developmental potential, polyspermy), hormonal imbalance (progesterone deficiency, abnormal PGF₂*α* induction), changes in the uterine microenvironment, as well as reproductive disorders such as embryo loss, fetal growth restriction, placental malformation, and even miscarriage (as shown in Figures 5a–f, the multi-dimensional damage to follicles, corpus luteum, oocytes, and pregnancy stages) (25).

**Figure 6:**
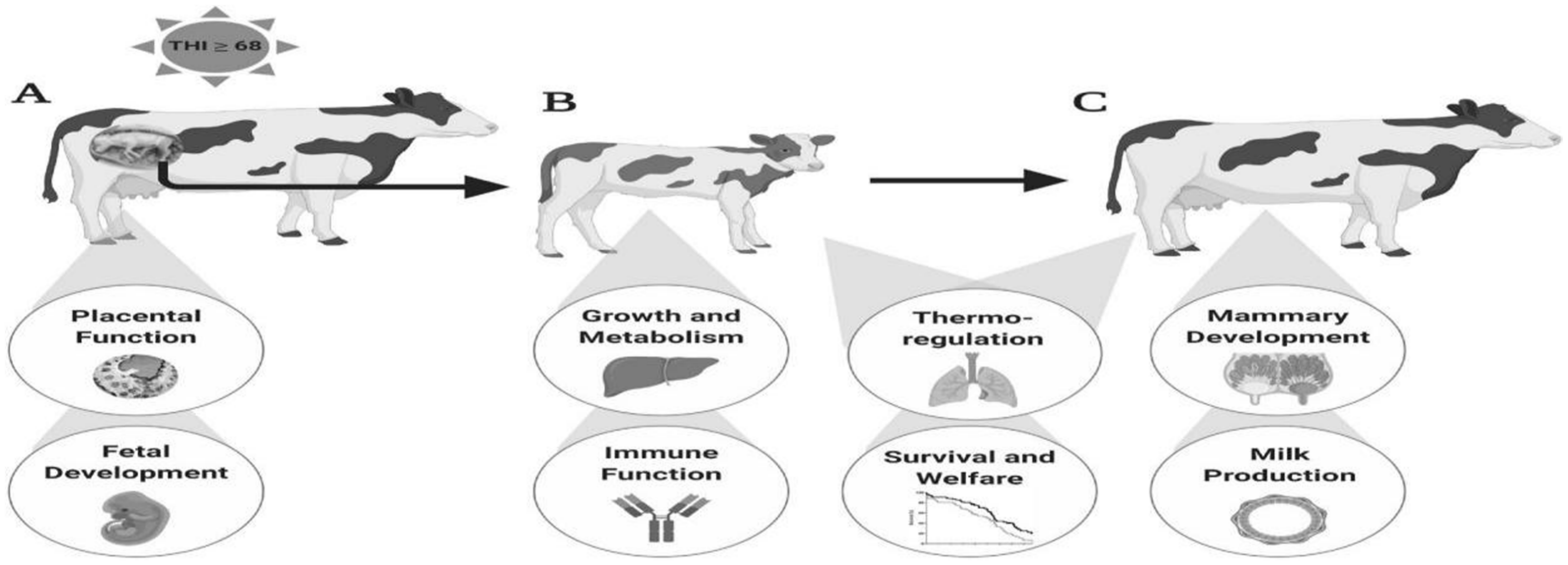
Summary of impacts of intrauterine heat stress on offspring outcomes: **(A)** developing fetus, **(B)** postpartum calf, and **(C)** mature cow. When the Temperature—Humidity Index is ≥ 68, it affects the physiological functions of cows at various life stages, from pregnant cows to calves and then to adult cows (such as placental function, fetal development, growth and metabolism, immunity, thermoregulation, mammary gland development, milk production, etc.).

Persistent transgenerational effects: Intrauterine heat stress damages placental and fetal development during the fetal period, interferes with growth metabolism (e.g., liver function) and immune function during the juvenile period, and affects thermoregulation, mammary development, and lactation performance during adulthood (as shown in Figures 6A–C, the long-term shaping of offspring’s life course from “fetus → calf → adult cow”), comprehensively impairing dairy cow reproductive efficiency and offspring quality (60).

### 3.1 Damage to oocyte and follicle development caused by heat stress

Heat stress is associated with an increased abortion rate of early embryos (124). Perinatal nutritional imbalance further exacerbates reproductive disorders (63). Furthermore, the damage of heat stress to oocytes persists throughout the oocyte developmental cycle (64, 65).

During the follicular phase, high temperatures inhibit granulosa cell proliferation, reducing the expression of follicle-stimulating hormone (FSH) receptors by 30–40% (relative decrease) and hindering follicle development (25). *In vitro* studies have shown that after 24 h of treatment at 39 °C, oocyte mitochondrial membrane potential decreases by 25% (relative decrease), adenosine triphosphate (ATP) production reduces by 40% (relative decrease), and the incidence of spindle abnormalities increases from 5 to 22% (absolute proportion) (66). Transcriptome analysis reveals that in heat-stressed oocytes, genes related to cell apoptosis (Bax and Caspase3) are upregulated, while genes related to antioxidant activity (SOD1, superoxide dismutase 1; CAT, catalase) are downregulated, with a twofold increase in DNA breakage rate (48).

### 3.2 Impacts of heat stress on bull reproductive function

Bull testes are sensitive to temperature; an increase in scrotal temperature to 23 °C affects spermatogenesis. Heat stress rapidly increases sperm abnormality rate [from 15 to 30% (absolute proportion)], reduces motility [motility < 50% (absolute proportion)], and impairs acrosome integrity (67). Proteomic studies indicate that HSP90 expression increases in heat-stressed sperm, while the activity of antioxidant enzymes (e.g., glutathione peroxidase) decreases by 20–30% (relative decrease), exacerbating lipid peroxidation damage (68).

### 3.3 Interference of hormonal imbalance on reproductive processes

High temperatures inhibit the pulsatile frequency of gonadotropin-releasing hormone (GnRH) in the hypothalamus, reducing the peak level of luteinizing hormone (LH) by 15–20% (relative decrease) and delaying the secretion of estrogen (E2) (69). Behavioral observations show that heat stress shortens the estrous duration of dairy cows from 18 to 12 h, and the proportion of silent estrus rises from 20 to 40% (absolute proportion) (25). This makes it difficult to determine the optimal timing for artificial insemination, thereby affecting the conception rate of dairy cows (70).

### 3.4 Changes in uterine microenvironment and embryo implantation

Heat stress can affect embryo implantation by altering the intrauterine microenvironment. It significantly impacts the estrous cycle and embryo implantation of dairy cows, leading to decreased conception rates (71). Studies have shown that endometrial cells exposed to 41 °C secrete 50% more (relative increase) prostaglandin (PGF2α) and express 30% less (relative decrease) leukemia inhibitory factor (LIF), thereby reducing the adhesion capacity of trophoblast cells (72). Clinical data indicate that embryo implantation success rates are 15–20% lower (relative decrease) in summer compared to winter (26).

### 3.5 Impacts of heat stress on fetal development

Heat stress in late pregnancy affects fetal development through multiple mechanisms. On one hand, it causes placental vasoconstriction and abnormal blood flow distribution, reducing fetal nutrient supply, impairing placental function [e.g., 20% weight loss (relative decrease)], lowering fetal growth rate to 0.40 kg/d, shortening gestation by 2 days, and reducing birth weight by approximately 4 kg (equivalent to 9% of normal weight) (73, 74). On the other hand, abnormal placental morphology (e.g., 1.5-fold increase in teratoma incidence, 15% abnormal expansion (relative increase) of cotyledon surface area) further reduces nutrient transport capacity (75). Simultaneously, the level of key placental hormone estradiol sulfate decreases by more than 30% (relative decrease) (76), collectively leading to intrauterine growth restriction of the fetus (77).

In addition, intrauterine heat stress causes abnormal DNA methylation (e.g., increased methylation levels in the IGF1 gene promoter region), resulting in poor mammary development in offspring when they reach adulthood [20% fewer alveoli (relative decrease)] and 8–12% lower milk yield (relative decrease) (epigenetic impacts of heat stress on offspring production potential, see section 4.3) (47). The impact pathway can be summarized as: prenatal heat stress (THI > 78) leads to placental dysfunction (reduced blood flow, nutrient deficiency), triggering fetal epigenetic changes (e.g., IGF1 gene methylation), ultimately resulting in poor mammary development and reduced milk yield in adulthood (76).

## 4 Impacts of heat stress on dairy cow production performance

The detrimental effects of heat stress on dairy cows are systemic and multi-targeted (17). Its negative impacts not only profoundly affect the reproductive system, interfering with gamete formation, embryo development, and hormonal balance, leading to a significant decline in reproductive efficiency, but this systemic physiological disorder also spreads to production-related core functions, exerting equally significant and complex impacts on dairy cows’ milk production performance (1). Next, we will specifically elaborate on how heat stress affects milk yield, milk composition, and long-term production potential of dairy cows by influencing feed intake, mammary function, and energy metabolism (2).

### 4.1 Direct impact of heat stress on milk yield

Studies have shown that heat stress significantly impairs dairy cows’ physiological functions, milk yield, and milk quality (78). When THI exceeds 72, the downward trend in milk yield aligns with the performance of moderate heat stress (see section 1): daily milk yield decreases by 1.3–6.7 kg/d, and total summer milk yield is 15–25% lower (relative decrease) than in winter (27). This is because high temperatures reduce rumen motility frequency by 20% (relative decrease) and suppress the appetite center, leading to a 10–15% decrease (relative decrease) in DMI (6). Mechanistically, high temperatures induce apoptosis of mammary epithelial cells, lowering the expression of key milk protein synthesis genes (e.g., *β*-lactoglobulin, *α*-casein) by 30–40% (relative decrease) (79). The body allocates energy preferentially to heat dissipation to maintain thermal homeostasis, reducing the energy available for lactation by 10–20% (relative decrease) (61). Further studies have shown that each 1-unit increase in THI significantly reduces DMI and energy-corrected milk (ECM) yield in mid-lactation cows by 4.13 and 3.25% (relative decrease), respectively (62).

### 4.2 Deterioration of milk composition and quality

Heat stress also alters milk composition and quality (80). Specifically, milk protein content decreases, while somatic cell count (SCC) increases significantly (81). This is because at 40 °C, the phagocytic activity of neutrophils decreases by 40% (relative decrease), impairing the mammary gland’s defense capacity and thus increasing SCC (81). Additionally, the biological activity of fat synthesis-related enzymes (e.g., fatty acid synthase, FAS) decreases by 15–20% (relative decrease), while the concentration of oxidative by-products (e.g., malondialdehyde, MDA) in milk doubles. Simultaneously, for every 0.30 percentage point decrease in milk fat content, milk protein content decreases by 0.20 percentage points (82). These changes affect the processing characteristics and quality of milk (83).

### 4.3 Transgenerational impacts of heat stress on production performance

In addition to reproductive performance, transgenerational impacts of heat stress are also reflected in production performance, with consequences involving not only immediate yield losses but also potential reshaping of the long-term production potential of dairy herds through epigenetic mechanisms (84). During the first lactation period, heat stress reduces daily milk yield by 3–5 kg/d, equivalent to 15–20% of the standard yield; simultaneously, the synthesis of milk fat, milk protein, and lactose decreases by 18, 15, and 12% (relative decrease), respectively (60, 85). This decline in production performance accumulates over multiple lactation cycles.

In-depth exploration of the mechanisms reveals that abnormal mammary development caused by heat stress results from both structural developmental defects and abnormal epigenetic regulation (60). Structurally, mammary alveoli volume decreases, epithelial cell proliferation rate declines, and damage to mammary structure from extreme heat stress can persist in offspring. At the epigenetic level, 327 genes involved in cell proliferation, immune, and metabolic pathways exhibit differential methylation, among which the methylation level of genes related to the Wnt/β-catenin signaling pathway doubles (86). These molecular changes may continuously affect the functional differentiation of mammary tissue through microRNA (miRNA) regulatory networks, ultimately leading to permanent impairment of milk production capacity (48). Therefore, the impacts of heat stress are persistent and cumulative. It is not only a direct cause of current declines in production performance but also poses a potential threat to the long-term production potential of dairy herds, requiring urgent attention and in-depth research on mitigation strategies (60). Specific data on the impacts of heat stress on dairy cow production performance are shown in Table 2.

**Table 2:** Effects of heat stress on dairy cow production performance.

Indicator	Normal conditions (THI < 68)	Heat stress conditions (THI >72) (absolute value)	Variation range (relative value)	References
Milk yield	Stable, no significant fluctuation	Daily milk yield reduced by 1.3–6.7 kg/d	↓15–25%	(27)
Milk protein percentage	2.8–3.2%	Decreased by 0.2–0.3%	↓7–10%	(82)
Milk fat percentage	3.5–4.0%	Decreased by 0.3–0.5%	↓8–13%	(82)
Somatic cell count (SCC)	<200,000 cells/mL	>500,000 cells/mL	↑Over 150%	(82)
Dry matter intake (DMI)	20–25 kg/d	Decreased 2–3.75 kg/d	↓10–15%	(27)

In summary, as a core challenge facing the dairy industry under global warming, heat stress exhibits multi-dimensional and cross-system complexity: at the reproductive level, it disrupts oocyte development, hormonal balance, and uterine microenvironment, leading to decreased conception rates, reduced embryo survival rates, and even transgenerational reproductive damage; at the production performance level, it causes reduced feed intake, impaired mammary function, significant declines in milk yield and quality, and affects the long-term production potential of offspring through epigenetic mechanisms; while its underlying physiological mechanisms involve a series of cascading reactions such as oxidative stress imbalance, HPA axis overactivation, cellular structural and functional damage, altered gene expression patterns, and disrupted host-gut microbiota interactions (17). These multi-targeted negative impacts interact, not only threatening dairy cow health and welfare but also posing severe challenges to the economic sustainability of the industry (1). Based on a systematic analysis of the pathways and hazards of heat stress, the following will focus on interdisciplinary mitigation strategies, exploring how to scientifically construct a heat stress response system from the perspectives of environmental regulation, nutritional intervention, and genetic improvement, providing practical solutions for the dairy industry to address climate challenges.

## 5 Strategies to mitigate heat stress

### 5.1 Environmental optimization and physical cooling strategies

One of the key measures to mitigate heat stress is to improve farm environmental conditions (87). Specifically, environmental temperature and humidity can be reduced by providing adequate ventilation and shading facilities, as well as using cooling equipment such as spray systems and fans (88). Reasonably designed barns can also effectively alleviate the impacts of heat stress (89). In modern farm heat stress prevention and control systems, combining physical environmental modification with refined management measures has shown significant effects (90). In terms of barn environmental optimization, the composite cooling network composed of longitudinal ventilation and high-pressure atomization systems (droplet diameter ≤ 50 μm) has been under pilot application in some intensive domestic dairy farms. It achieves efficient cooling through pulsed cooling (activated for 30s every 5–10 min) while preventing excessive humidity, and this mode also reduces water consumption (91). In tropical regions with strong radiant heat, tunnel-type negative pressure ventilation systems can accelerate surface evaporative heat dissipation through cross-ventilation of ≥2 m/s, combined with nano-level reflective sunshades with 70–80% shading rate (≥95% ultraviolet blocking rate), which can continuously reduce cow rectal temperature (RT) by 0.5–1.0 °C, maintaining heat balance even under extreme conditions with an average daily temperature exceeding 35 °C (92).

### 5.2 Three-dimensional regulatory system of nutritional intervention

Nutritional management plays a key role in alleviating heat stress responses in dairy cows (93). Adjusting feed formulas to increase energy, protein, and mineral content, while supplementing appropriate vitamins and trace elements, can enhance dairy cows’ heat stress resistance (94). Studies have shown that supplementing a certain amount of antioxidants under heat stress conditions can reduce oxidative stress in dairy cows and improve production performance (95). In response to the challenge of extreme high temperatures in the summer of 2025, innovative nutritional management has become a core technical means to address heat stress in dairy cows. The three-dimensional nutritional intervention system consisting of “antioxidant defense, energy metabolism optimization, and protein homeostasis regulation” can achieve the dual goals of protecting physiological functions and maintaining production performance (96).

In terms of strengthening the antioxidant system, combined intake of vitamin E (800–1,000 IU/d) and selenium (0.3–0.5 mg/kg feed) can reduce lipid peroxidation product (MDA) levels by 15–20% (relative decrease) and increase glutathione peroxidase (GSH-Px) activity by 25% (relative increase), forming the first antioxidant defense line (6). Meanwhile, flavonoids such as dihydromyricetin can target and inhibit mitochondrial fission protein Drp1, reducing mammary cell apoptosis caused by heat damage by 30% (relative decrease) (97). Introducing functional additives such as melatonin (10–20 mg/day) can not only correct circadian rhythm disorders caused by high temperatures but also improve oocyte maturation rate by reducing cortisol levels by 10–15% (relative decrease); combined with nicotinamide mononucleotide (NMN, a direct precursor of coenzyme nicotinamide adenine dinucleotide (NAD+) involved in energy metabolism and other physiological processes) that repairs mitochondrial complex I, it can increase ATP production efficiency in mammary cells by 30% (relative increase), providing continuous energy support for milk component synthesis (31).

In terms of energy and protein metabolism regulation, *ω*-3 polyunsaturated fatty acids (e.g., 30 g/day fish oil) can significantly reduce the secretion of inflammatory factors such as IL-6 and TNF-*α* by 40% by inhibiting the nuclear factor kappa-light-chain-enhancer of activated B cells (NF-κB) signaling pathway (6). Simultaneously, precise supplementation of rumen-protected methionine (20 g/d) can increase milk protein synthesis efficiency by 12–15%, effectively reversing nitrogen imbalance caused by heat stress (98). To address the key issue of electrolyte imbalance, a scientific ratio of 1.5–2.0% potassium chloride and 0.8–1.0% sodium bicarbonate added to the diet can maintain blood pH within the physiological range of 7.35–7.45, while improving rumen buffering capacity and increasing DMI by 8–10% (relative increase) (27). This solution was validated during the 2024 heat stress peak in northern China (THI 82 for 15 consecutive days), successfully maintaining the average daily milk yield of lactating cows at the industry-leading level of 28–30 kg/d (27). Current ranch management systems integrate these parameters into intelligent feeding terminals, allowing for dynamic adjustments to nutritional supply plans based on real-time temperature and humidity data (6). The system establishes a closed-loop management process including “stress early warning, nutritional intervention, and effect evaluation” (27).

### 5.3 Genetic breeding and heat tolerance improvement

In the context of accelerating global warming, the deep integration of genetic selection and molecular breeding technologies is becoming a strategic means to improve the heat stress adaptability of dairy cows (99, 100). By systematically screening individuals with heat-tolerant genes and utilizing heterosis, a new breeding model that optimizes interactions among genotype, phenotype, and environment is emerging (101). At the breed improvement level, crossing tropical breeds such as Brahman cattle (carrying the melanocortin 1 receptor (MC1R) gene) and Zebu cattle (with high heat shock protein beta-1 (HSPB1) gene expression) with Holstein cattle results in F1 offspring with a respiratory rate 15 breaths/min lower than purebred Holstein cattle under THI 80, while increasing average daily milk yield by 10–15% (relative increase) (102). This phenotypic advantage stems from the upregulated expression of heat shock proteins (e.g., HSP70) and enhanced mitochondrial function stability conferred by heat-tolerant genes (103).

Molecular marker-assisted selection technology further accelerates the breeding process (102). Based on genome-wide association studies (GWAS), two key gene loci related to heat tolerance have been precisely located: BRWD1 gene cluster (bovine chromosome 1, BTA1): Full name Bromodomain and WD Repeat Domain Containing 1, a key regulator of HSP70 synthesis. EXD2 gene (chromosome 10 BTA10): 3′–5′ exonuclease domain protein 2, a protein-coding gene significant in dairy cow heat stress response (102). It reduces DNA heat damage rate through the base excision repair pathway and plays multiple key roles in cellular processes (102). Combined with the single-step genomic best linear unbiased prediction (ssGBLUP) algorithm integrating pedigree and genomic information, the prediction accuracy of heat tolerance trait breeding values estimated breeding values (EBV) has increased by 20–30% (relative increase) (51). This method was successfully applied at the University of Florida Experimental Station in 2024, reducing heat stress-related milk yield decline in the core breeding population by 18% (relative decrease) (67). Notably, the International Committee for Animal Recording (ICAR) has incorporated these molecular markers into the global dairy cattle genetic evaluation system, promoting the cross-regional spread of heat-tolerant genes through cross-border circulation of frozen semen (102). It is expected that by 2026, this method will cover 30% of the global high-yielding dairy cattle population (102).

### 5.4 Synergistic innovation in reproductive management and immune regulation

In high-temperature and high-humidity environments, synergistic innovation in reproductive management and immune regulation has become a necessary means to ensure dairy cow reproductive efficiency (51). To address the increased follicular atresia rate caused by heat stress, the optimized timed artificial insemination (TAI) protocol uses a three-injection method of GnRH (gonadotropin-releasing hormone), PGF2α (prostaglandin), and GnRH at 7-day intervals, combined with a progesterone-releasing intravaginal device (controlled internal drug release, CIDR) containing 1.38 g progesterone (104). This method can precisely regulate the synchronization of corpus luteum formation and follicle development, increasing the conception rate from 25 to 37–40% (absolute proportion), and maintaining a stable pregnancy rate of over 32% (absolute proportion) even under continuous THI > 78 (68).

Another breakthrough technology is vaccinating dairy cows with inhibin α subunit vaccine (2 mL per head per dose, containing 200 μg antigen), which can neutralize over 70% (relative decrease) of serum inhibin concentration, increase follicle-stimulating hormone (FSH) levels by 30% (relative increase), and make dominant follicles exceed 18–20 mm in diameter (104). When combined with the progesterone slow-release system, it can increase the conception rate of high-yielding dairy cows to over 40% (absolute proportion) (see Table 3 for specific effect comparison), with ovulation synchronization rate 2–3 times higher than traditional protocols (104). In the field of immune enhancement, the gut-mammary axis bidirectional regulation strategy is effective: supplementing *Bacillus subtilis* can colonize the terminal ileum, inhibiting the growth of harmful bacteria such as *Escherichia coli* O157: H7, reducing plasma endotoxin (LPS) concentration by 50% and inflammatory factor IL-6 level by 20% (105). Simultaneous use of astragalus polysaccharides (20 g/d) can activate the Nrf2/antioxidant response element (ARE) pathway, increasing superoxide dismutase (SOD) and catalase (CAT) activities by 15–20% (relative increase), forming a dual defense barrier against oxidative stress and inflammatory responses (106). During the 2024 summer high-temperature period in the Yangtze River Basin (28 consecutive days with THI 82), this system was verified to successfully reduce the incidence of dairy cow mastitis from 18.7 to 9.2% (absolute proportion) while maintaining milk fat rate above 3.8% (absolute proportion) (105). Current farm management systems have integrated a meteorological early warning module, which automatically activates the above regulatory protocols when THI exceeds 75, realizing an intelligent closed-loop management of heat stress prevention and control (21).

**Table 3:** Comparison of heat stress mitigation strategies and their effects.

Strategy type	Specific measures	Effects	Feasibility	References
Environmental control	Longitudinal ventilation + high-pressure atomization (droplet ≤ 50 μm)	Indoor temperature ↓3–5 °C, THI ↓8–10 units	High technical threshold; in experimental stage, requiring long-term validation	(87)
	Negative pressure tunnel ventilation + reflective sunshade net (shading rate 70–80%)	Rectal temperature ↓0.5–1.0 °C	Easy to operate; can be directly installed in existing barns, compatible with existing breeding processes	(87)
Nutritional intervention	Vitamin E (800–1,000 IU/day) + Selenium (dosage see section 5.2)	MDA ↓15–20%, GSH-Px activity ↑25% (relative value)	Requires sensors, algorithms, and data support; suitable for large-scale pastures	(6, 93)
	Melatonin (10–20 mg/day) + NMN (dosage see section 5.2)	Cortisol ↓10–15%, ATP production ↑30% (relative value)	Mature technology; easy to install and maintain, compatible with pastures in tropical, subtropical, and temperate regions	(27)
Genetic breeding	Holstein × Brahman (MC1R gene)	Respiratory rate ↓15 breaths/min, milk yield ↑10–15% (relative value)	Feasible	(102)
	Genome-wide selection (BRWD1, EXD2 gene markers)	Prediction accuracy of heat tolerance EBV ↑20–30% (relative value)	High technical threshold; in experimental stage, requiring long-term validation	(102)
Smart monitoring	Infrared thermography (parameters see section 5.5)	Heat stress early warning 2–3 h in advance, accuracy 92%	Easy to operate; can be directly used in herd heat stress monitoring, compatible with existing breeding processes	(34)
	SMARTBOW ear tag (parameters see section 5.5)	Milk yield volatility of sensitive groups reduced from ±15 to ±5% (relative coefficient of variation)	Requires sensors, algorithms, and data support; suitable for large-scale pastures	(107)

### 5.5 Application of intelligent monitoring and early warning systems

**Figure 7:**
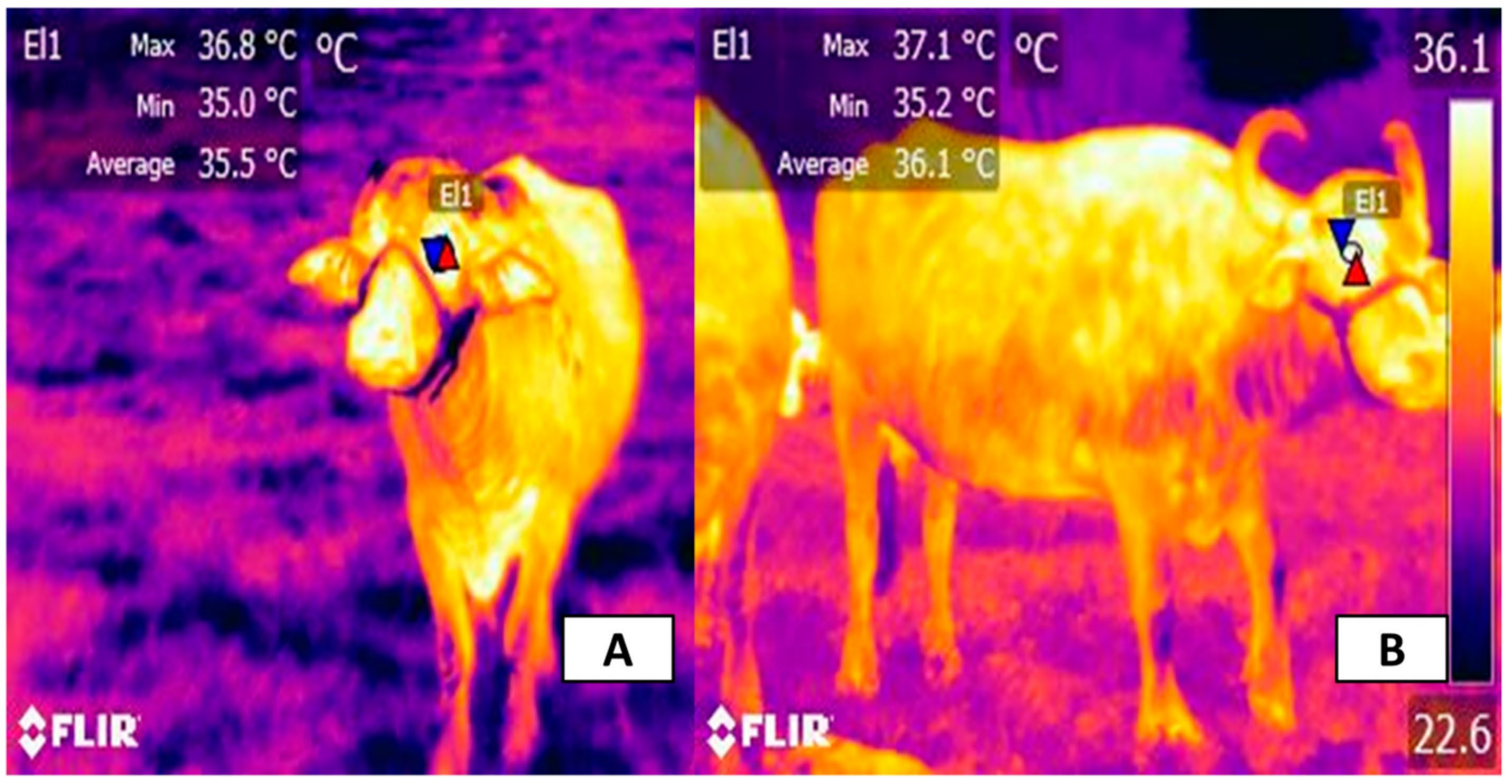
Application of infrared thermography in evaluating thermal responses of farm animals under heat stress. Comparison of body temperatures of two animals detected by FLIR thermal imaging technology: In **(A)**, the maximum temperature in the detection area is 36.8 °C, the minimum is 35.0 °C, and the average is 35.5 °C; in **(B)**, the maximum temperature in the detection area is 37.1 °C, the minimum is 35.2 °C, and the average is 36.1 °C. The color difference reflects the body surface temperature distribution and overall body temperature level.

Infrared thermography (IRT) can detect microvascular dilation signals around cows’ ears, mouths, noses, and eye sockets, with a temperature sensitivity of ±0.05 °C, enabling early warning of heat stress 2–3 h in advance with 92% accuracy (absolute accuracy), as shown in Figure 7 (34). The intelligent early warning system based on multi-modal data fusion has two core functions: Data integration: Dynamically integrates five key parameters—THI, respiratory rate (RR), rectal temperature (RT), behavioral parameters (e.g., lying time), and activity level (e.g., step frequency). Real-time calculation: Uses a random forest model to compute the heat stress index, with feature weights distributed as follows: THI (35%), RR (25%), RT (20%), lying time (15%), and activity level (5%) (21). When the index exceeds 72, hierarchical responses are automatically activated: level 1 warning (THI > 72) activates longitudinal ventilation; level 2 response (RR > 60 breaths/min) activates spray cooling; emergency intervention (RT > 39.5 °C) triggers combined cooling with water curtains and air conditioning, reducing heat exposure time by 40% (21).

To address individual differences, the SMARTBOW ear tag system uploads core body temperature and activity data every 10 min, and through clustering algorithms, prioritizes allocating early lactation cows (postpartum < 100 d) and high-yielding cows (daily milk yield > 35 kg/d) to cooling areas, reducing the coefficient of variation of daily milk yield in sensitive groups from ±15 to ±5% (relative coefficient of variation) in summer (107). Infrared thermography has a sensitivity of ±0.05 °C and an early warning accuracy of 92%, which is affected by sunlight, humidity, and individual cow differences and needs regular calibration with rectal temperature; SMARTBOW ear tags transmit data every 10 min, which can reduce milk yield volatility in sensitive groups, and are affected by high temperature and humidity and wearing conditions, requiring calibration to ensure data accuracy (34). It is worth noting that chronic heat stress (THI 75–85 for more than 30 consecutive d) can cause irreversible damage, including a 15–20% reduction (relative decrease) in mammary alveoli density and a 30% increase (relative increase) in mammary duct dilation (79). Even if the environment improves later, milk production performance can only recover to 80–85% of the pre-stress level (relative recovery) (108). More seriously, heat exposure during pregnancy can affect offspring through epigenetic mechanisms: the methylation level of the IGF1 gene promoter region in the offspring’s liver increases by 12–18% (relative increase), leading to impaired glucose tolerance in adulthood (10–15% (relative decrease) reduction in the area under the oral glucose tolerance test (OGTT) curve) (60). This metabolic abnormality can persist into the third generation, indicating that heat stress is changing the evolutionary trajectory of dairy cow populations (109). A comparison of specific heat stress mitigation measures and their effects is shown in Table 3.

Current artificial intelligence technologies provide comprehensive solutions for dairy cow heat stress management (110). At the monitoring level, intelligent systems use You Only Look Once Version 5 (YOLOv5) algorithm and Canny edge detection technology to identify cow chest and abdomen behaviors. The system judges the degree of heat stress in real-time by analyzing parameters such as respiratory rate and body temperature fluctuations. Combined with the respiratory-temperature correlation model, precise classification (moderate/severe) can be achieved (97). In addition, the multi-modal data fusion system integrates infrared temperature measurement, heart rate monitoring, and meteorological parameters such as temperature and humidity to build a machine learning prediction model, significantly improving the accuracy of risk early warning (111).

In terms of environmental regulation, the THI early warning system developed by Mengniu Group can connect to weather forecast interfaces. The combination of intelligent spray equipment and ventilation can increase milk yield by 19.8%, and the system can dynamically adjust the microclimate in the barn (112). In the drinking and feeding link, the sensor network ensures that each cow meets the daily water intake of 140 kg. Meanwhile, ice water supply combined with dynamic formula adjustment adding vitamin E and *β*-carotene forms a dual cooling mechanism (6).

In health management, artificial intelligence provides early warning of diseases such as mastitis by analyzing behavioral abnormalities such as standing time and feed intake, as well as changes in somatic cell count (21). Despite challenges such as data standardization and model generalization, future research will focus on breakthroughs in multiple fields, such as screening heat-tolerant dairy cows through gene editing (e.g., S441 marker), building industry-level edge computing platforms, and realizing multi-ranch data sharing (102). This will promote the development of intelligent and precise heat stress management.

### 5.6 Synergistic effects and adaptability analysis of multiple strategies

The synergistic application of different mitigation strategies can amplify effects through functional complementarity, with comprehensive benefits significantly better than single measures, and adaptability adjustments based on breed characteristics and climate regional differences are needed, as follows:

#### 5.6.1 Closed-loop synergy between intelligent monitoring and environmental/nutritional intervention

Real-time linkage between intelligent monitoring systems (e.g., infrared thermography, SMARTBOW ear tags) and environmental optimization, nutritional regulation can form an efficient “perception-decision-execution” closed loop (81). For example, when the intelligent system detects THI rising to 72 (level 1 warning), it automatically activates longitudinal ventilation + high-pressure spray (environmental optimization), and simultaneously increases the supplementation of selenium-vitamin E in the diet through intelligent feeding terminals (nutritional intervention) (6). This combination can increase milk yield by an additional 8–10% (relative increase) compared to single environmental cooling, and reduce the decline in milk protein rate by 0.1–0.15 percentage points (106). The “THI warning + dynamic spray + intelligent nutritional adjustment” system increases summer milk yield by 19.8% (relative increase) compared to using spray equipment alone, confirming the advantages of synergistic regulation (110).

#### 5.6.2 Adaptability optimization between genetic breeding and environmental regulation

The breeding of heat-tolerant breeds can reduce dependence on environmental regulation, but management strategies need to be adjusted according to climate regional characteristics (25, 53). For example, crossbred offspring of Brahman and Holstein cattle (carrying the MC1R gene) can reduce the operating time of cooling equipment by 30% (relative decrease) in tropical regions (THI 80), while in temperate regions (seasonal THI fluctuation 60–85), it is necessary to combine winter insulation measures (e.g., increasing bedding thickness to 8 cm) to balance their insufficient cold tolerance (102). Studies have shown that tropical crossbred breeds have 10–15% higher (relative increase) milk yield than purebred Holstein cattle in summer in temperate regions, but require maintaining environmental temperature at 15–20 °C in winter to avoid a 5–8% (relative decrease) decrease in milk yield (53).

#### 5.6.3 Synergistic enhancement of nutritional intervention and reproductive management

The combination of antioxidant nutrition and reproductive regulation technology can significantly improve reproductive efficiency under heat stress (104). For example, supplementing melatonin (10–20 mg/d) synchronously in the timed artificial insemination (TAI) protocol can increase the conception rate by 7–9 percentage points compared to TAI alone, and reduce oocyte apoptosis rate by 12% (relative decrease) (103). Practice in the Yangtze River Basin shows that the combination of “*Bacillus subtilis* (intestinal immune regulation) + progesterone slow-release device (reproductive management)” reduces the incidence of mastitis from 18.7 to 9.2% (absolute proportion) at THI 82, while maintaining the conception rate above 37% (absolute proportion), 12% higher (relative increase) than single reproductive management (105).

#### 5.6.4 Cost–benefit advantages of cross-strategy synergy

The input–output ratio of combined strategies is significantly higher than that of single measures. For example, the low-cost combination of “sunshade net (cost < 10,000 yuan/ha) + selenium-vitamin E supplementation (daily cost < 2 yuan/head.d)” can reduce losses by an additional 5–8% (relative decrease) compared to using sunshade nets alone, while the high-end combination of “intelligent monitoring + precise cooling + targeted nutrition” has an initial investment increase of 30% (relative increase), but the increase in milk yield and reduction in medical costs can shorten the investment payback period to 1.5–2 years (113).

In summary, the synergy of multiple strategies can maximize the efficiency of heat stress prevention and control through multi-dimensional linkage of “monitoring and prediction—precise regulation—breed adaptation—nutritional support (27). In the future, it is necessary to establish adaptation parameter models of breed-environment-nutrition for different climate regions (e.g., arid high-temperature regions, high-humidity temperate regions) to provide quantitative basis for differentiated management (102).

#### 5.6.5 Core application points—a practical guide for farmers and veterinarians on heat stress prevention and control

In terms of environmental regulation, overhaul sunshade nets (with a shading rate of 70–80%) before summer (92). During high-temperature periods (10:00–16:00), use the “fan + spray” combination for cooling (30 s on, 5–10 min off)—this combined cooling strategy has been proven to reduce cow body temperature by 0.8–1.2 °C and improve milk yield retention by 12–15% compared to single cooling methods (14). For tropical/subtropical pastures, add tunnel-type negative pressure ventilation, which can lower barn temperature by 4–5 °C and reduce rectal temperature of lactating cows by 0.6–0.8 °C under THI > 80 (92); for temperate pastures experiencing short-term heat stress, strengthen sprinkling and manure cleaning—frequent sprinkling (once every 2 h) combined with daily manure removal can reduce barn heat load by 12–15% (relative decrease) (91).

For nutritional supplementation, when THI > 72, supplement the entire herd with vitamin E (800–1,000 IU/d) and selenium (0.3–0.5 mg/kg feed), and add electrolytes to drinking water—this combination increases plasma glutathione peroxidase activity by 28% and reduces malondialdehyde levels by 21% (relative decrease), alleviating oxidative damage (59). High-yield cows should receive additional fish oil supplementation (30–40 g per cow per day), which inhibits the NF-κB pathway to reduce inflammatory factor secretion by 35% (relative decrease) (4), while weak or sick cows can be given astragalus polysaccharides (20–30 g per cow per day)—similar to plant polyphenols, it activates the Nrf2/ARE pathway to enhance antioxidant capacity (96).

In monitoring, farmers should observe cow behaviors (intervention is needed if over 30% of cows show open-mouth breathing or stand for more than 8 h) and measure respiratory rates (alert if over 60 breaths per minute)—these behavioral indicators have a 0.85 correlation with heat stress severity (6). Veterinarians should intervene based on THI classification (mild stress: rectal temperature 38.6–39.5 °C; severe stress: rectal temperature > 40 °C)—this classification has 92% accuracy in judging stress levels (13).

For reproductive management, avoid breeding during high-temperature periods (THI > 72)—conception rates decrease by 28–32%(relative decrease) when breeding at THI 75–80 (25). For pregnant cows after insemination, inject progesterone (50–100 mg per cow) to maintain pregnancy when THI > 78—this measure increases embryo survival rate by 20–23% (104).

In emergency response, when THI > 80, spray the neck and abdomen of cows with normal temperature water (15–20 °C) and feed warm sugar water (5–8% concentration)—warm sugar water replenishes glycogen to reduce ketone bodies by 22%(relative decrease) within 3 h (61). Veterinarians should inject mannitol (0.5–1 g/kg body weight) into severely affected cows, and check for mastitis and intestinal health in the later stage—heat stress increases mastitis incidence by 18–20%, requiring somatic cell count monitoring (99).

Pastures of different scales can adapt corresponding measures according to their conditions to reduce losses in dairy cow performance—large-scale farms benefit from intelligent ventilation, while small-scale farms prioritize low-cost sunshade and basic nutrition (105).

## 6 Conclusion and prospects

### 6.1 Key gaps in research on the impacts of heat stress on dairy cows

Based on the current research progress on the impacts of heat stress on dairy cows and the practical needs of the industry, although existing studies have covered physiological mechanisms such as oxidative stress imbalance and overactivation of the hypothalamic–pituitary–adrenal (HPA) axis, and have also developed practical mitigation strategies including environmental optimization and nutritional regulation—providing important theoretical support and technical references for the dairy industry—there are still several key research gaps in the field from the perspectives of practical management needs of livestock farms and research priorities of researchers (86). These gaps not only limit the comprehensive understanding of the complexity of heat stress in dairy cows, but also make it difficult for livestock farms to implement more precise and efficient prevention and control solutions, and hinder researchers from breaking through technical bottlenecks to build a sustainable prevention and control system (57).

From the perspective of daily management on livestock farms, behavioral changes in dairy cows under heat stress are the most observable signals for farm staff (15). But current research on interpreting and applying these signals still has clear limitations. In practice, farms can see phenomena like dairy cows’ daily lying time dropping to less than 12 h (24). Their feeding frequency falls by about 30% compared to normal levels. More than 30% of the herd also shows open-mouth breathing under heat stress (24). However, these observations mostly stay at the surface description level. Farms lack quantifiable indicators, such as the link between how long behavioral changes last and THI levels (15). They even cannot tell if dairy cows are in negative emotional states like anxiety or distress from their behaviors. For research, a correlation model of “specific heat stress conditions (e.g., different THI levels)—behavioral responses—emotional states” has not been built yet (114). This makes it hard to provide farms with welfare-focused management plans that consider both cows’ physical and mental health. In the end, this may affect cows’ long-term production performance and bring hidden economic losses to farms (24, 95).

At the same time, research on the combined effects of multiple environmental factors (like high temperature and humidity, air pollution) on dairy cows is insufficient. It cannot meet farms’ actual prevention needs, nor can it support breaking through the existing research framework. In real production, farms often face overlapping stressors. In intensive closed barns, heat stress may coexist with high-concentration ammonia pollution from manure (99). This threatens cows’ health. But most current studies treat heat stress as a single variable. They only explore how different THI levels affect milk composition and reproductive hormones (1). They have not systematically explained how multiple stressors work together. For example, they do not know if high humidity makes oxidative stress from heat stress worse. They also do not know if ammonia pollution weakens the immune system’s ability to tolerate heat stress. Besides, no practical comprehensive management strategies for combined stressors have been given to farms (10). So farms can only take “passive responses” when facing complex environments. This greatly reduces the effectiveness of prevention and control.

From the perspective of farms’ long-term development plans, heat stress’s impacts on the genetic potential of dairy cow groups and offspring’s production performance are crucial. But research on related mechanisms is still not enough. This is also a theoretical and technical difficulty that needs to be solved urgently. On one hand, the long-term results of heat stress and its transgenerational impact mechanisms are unclear. Farms know the immediate harms of heat stress, such as a daily milk yield reduction of 1.3–6.7 kg when THI ≥ 72 (27). But they do not understand its long-term impacts. For example, prenatal heat stress (THI ≥ 78 in late pregnancy) may make offspring have 20% fewer mammary alveoli when they grow up. Their milk yield may also drop by 8–12% (60). But farms cannot tell if this impact will pass to future generations. Existing studies only confirm that methylation of the IGF1 gene promoter may be involved in transgenerational impacts (60). Many key questions remain unanswered. Besides IGF1, which other key gene sites are regulated by prenatal heat stress? Are epigenetic modifications specific to certain tissues (2)? For example, do they only exist in mammary glands or also in the liver and reproductive system? How do these modifications achieve stable transgenerational inheritance? These unknowns stop research from providing farms with tools to assess the genetic risks of dairy cow groups. They also limit farms’ ability to make long-term breeding and population optimization plans (47).

On the other hand, the regulatory role of non-coding RNAs (ncRNAs) in heat stress is not fully explained. These include long non-coding RNAs (lncRNAs) and circular RNAs (circRNAs) (49). This not only affects the development of molecular-level technologies to improve heat tolerance (50). It also fails to provide farms with accurate early warning and breeding tools. Currently, researchers have found the roles of some ncRNAs. For example, the expression of miR-25 in the mammary tissue of heat-stressed cows increases (46). It makes mammary inflammation worse by targeting the JNK gene. LncRNAs may also regulate the transcription of the HSP70 gene (49). But this research is still in the preliminary stage and scattered. It has not clarified the complete regulatory network of ncRNAs. For example, it is unknown if circRNAs—acting as “miRNA sponges” that trap miR-181d—can ease immune disorders caused by heat stress (46). It also has not verified if ncRNAs can be used as heat stress biomarkers in large-scale field experiments (55). For example, can the expression level of miR-25 be used to assess the degree of mammary gland damage (46)? For farms, this means they cannot use molecular detection to warn of heat stress early. They also cannot breed heat-tolerant cows based on molecular markers. They can only rely on traditional prevention and control methods. The efficiency and accuracy of their prevention and control need to be improved.

### 6.2 Heat stress poses multi-dimensional threats to dairy cow welfare

Physiologically, increased rectal temperature (>40 °C) and sharply increased respiratory rate (>80 breaths/min) cause respiratory alkalosis and metabolic disorders, accompanied by discomfort such as open-mouth panting. Long-term high temperatures also increase the risk of diseases such as mastitis due to immunosuppression, further worsening such physiological discomfort (40); behaviorally, lying time is shortened to <12 h and feeding frequency is reduced by 30% (relative decrease), violating natural behavioral needs, and group aggregation may trigger aggressive behavior (115); psychologically, overactivation of the HPA axis increases cortisol levels by 1.8 times, inducing anxiety-like behaviors (e.g., frequent pacing) and causing persistent stress (38); reproductively, oocyte apoptosis rate rises to 22% (absolute proportion) and bull sperm motility drops below 50% (absolute proportion), causing reproductive disorders and indirectly increasing culling risk (60). Corresponding mitigation measures include: environmental optimization using pulsed spray combined with longitudinal ventilation (cooling by 3–5 °C), improving lying areas (≥5 cm thick bedding), and reasonably allocating space and resources (115); nutritional intervention supplementing melatonin (10–20 mg/d) and *ω*-3 polyunsaturated fatty acids (30 g/d fish oil) to reduce metabolic stress and pain (103); intelligent management monitoring behavioral abnormalities through infrared thermography [early warning 2–3 h in advance with 92% accuracy (absolute accuracy)] and SMARTBOW ear tags to trigger intervention (110); genetic breeding cultivating heat-tolerant crossbred breeds (e.g., Holstein × Brahman) to reduce respiratory rate by 15 breaths/min and reduce stress pain (102). It is worth noting that improved welfare can increase milk yield by 8–10% (relative increase) and reduce the decline in milk protein rate by 0.1–0.15 Percentage points, forming a positive cycle of “welfare-health-production.” In the future, it is necessary to further quantify cow emotional states and formulate welfare-based hierarchical management standards (106).

### 6.3 Key directions for future research

One of the key directions for future research is how to better apply precision livestock farming (PLF) technology to heat stress management (113). PLF is a breeding model based on modern information technology, data science, and intelligent equipment, aiming to improve livestock production efficiency, resource utilization, and animal welfare through real-time monitoring, precise regulation, and automated management (101). Meanwhile, under the background of climate change, studying the combined impacts of multiple environmental factors such as high temperature, high humidity, and air pollution on dairy cows and formulating more effective comprehensive management strategies are also of great significance (116).

The impact of heat stress on dairy cows is comprehensive, involving multiple targets, cross-systems, and long-term effects (117). Its negative impacts first manifest in the reproductive system, interfering with key processes such as gamete formation and embryo development, combined with the dual effects of hormonal imbalance and reproductive cell damage, ultimately leading to a significant decline in conception rate (86). At the same time, production performance is also subject to systemic inhibition, specifically manifested as insufficient nutrient intake due to reduced feed intake, lactation dysfunction caused by mammary tissue damage, and disruption of milk component synthesis pathways due to changes in energy metabolism (46). These factors collectively lead to continuous declines in milk yield and quality (118).

From a pathophysiological perspective, the three core molecular mechanisms of heat stress are:
Free radical accumulation caused by oxidative stress.
Hormonal imbalance caused by hypothalamic–pituitary–adrenal axis dysfunction.
Insufficient ATP synthesis caused by mitochondrial respiratory chain dysfunction (119).

In the field of animal husbandry research, heat stress-related research can be explored from multiple perspectives (58). To clarify precise mechanisms, it is necessary to conduct in-depth research on the regulatory role of non-coding RNAs [e.g., long non-coding RNAs (lncRNAs) and circular RNAs (circRNAs)] under heat stress to identify specific targets for epigenetic transgenerational transmission.

Non-coding RNAs (ncRNAs) represent a key future research direction: On one hand, it is necessary to supplement functional verification of dairy cow-specific ncRNAs—for example, clarifying lncRNAs’ regulatory mechanism on the heat shock protein 70 (HSP70) gene and verifying circRNAs’ role as ‘miRNA sponges’ in alleviating mammary inflammation (e.g., targeting miR-25) (49). This verification aims to improve the association chain from “molecular regulation → physiological response → production performance” (50). On the other hand, non-coding RNAs can be developed as biomarkers for heat tolerance (e.g., the association between miR-25 expression level and the degree of mammary gland damage) or used for precise regulation of reproductive capacity (e.g., improving mitochondrial function of oocytes under heat stress by regulating lncRNAs), providing new targets for genetic breeding and nutritional intervention (46). In addition, multi-omics analysis should be used to clarify the comprehensive role of genomics, proteomics, and metabolomics in heat stress adaptation, with particular attention to the gut-brain axis interaction and epigenetic memory mechanisms (120).

In developing efficient mitigation technologies, a synergistic intervention model combining environmental, nutritional, and genetic factors can be established. At the management level, an intelligent management system based on the Internet of Things (IoT) and artificial intelligence (AI) can be built to realize real-time monitoring and dynamic response to heat stress, thereby reducing labor costs and improving breeding efficiency (59).

In terms of economic impact assessment, it is necessary to establish regional and global scale economic loss models of heat stress (121). It is predicted that by 2,100, the global dairy industry will lose 40 billion US dollars annually due to heat stress. The model “global heat stress loss = basic loss × (1 + temperature rise coefficient)^n” can provide data support for policy-making and promote theoretical breakthroughs and practical applications of heat stress-related research (108).

### 6.4 Comprehensive strategies for heat stress management

In dairy cow heat stress management, short-term, medium-term, and long-term measures should be comprehensively implemented, supplemented by real-time early warning systems to improve animal welfare and production efficiency while reducing economic losses (122). In the short term, cooling equipment should be inspected before summer (see section 5.1 for specific equipment types and standards) to ensure that the coverage rate of fans and spray systems exceeds 90%, and the respiratory rate and rectal temperature of the herd should be regularly monitored. In the medium term, dairy cow diets should be optimized, antioxidant nutrients should be continuously supplemented (see section 5.2 for formula details), and files for heat-sensitive cows should be established. In the long term, genome selection technology should be used to breed heat-tolerant breeds (see section 5.3 for methods) to gradually improve the heat tolerance of the herd (123). In addition, by integrating real-time monitoring data and THI data can develop, a real-time early warning system can be developed to automatically activate spray and fan equipment, effectively reducing the adverse impacts of heat stress on dairy cows (107).
